# Global profiling of protein lipidation using chemical proteomic technologies

**DOI:** 10.1016/j.cbpa.2014.10.016

**Published:** 2015-02

**Authors:** Edward W Tate, Karunakaran A Kalesh, Thomas Lanyon-Hogg, Elisabeth M Storck, Emmanuelle Thinon

**Affiliations:** 1 Department of Chemistry, Imperial College London, Exhibition Road, London SW7 2AZ, UK; 2 The Rockefeller University, 1230 York Avenue, New York, USA

## Abstract

Protein lipidation is unique amongst post-translational modifications (PTMs) in enabling direct interaction with cell membranes, and is found in every form of life. Lipidation is important in normal function and in disease, but its intricate interplay with disease context presents a challenging for drug development. Global whole-proteome profiling of protein lipidation lies beyond the range of standard methods, but is well-suited to metabolic tagging with small ‘clickable’ chemical reporters that do not disrupt metabolism and function; chemoselective reactions are then used to add multifunctional labels exclusively to tagged-lipidated proteins. This chemical proteomic technology has opened up the first quantitative whole-proteome studies of the known major classes of protein lipidation, and the first insights into their full scope *in vivo*.

## Highlights

• Protein lipidation is an essential modification (PTM) in all forms of life.
• Key modifications include acylation, prenylation, cholesterylation and GPI anchors.
• Global profiling of this class of PTM is beyond the scope of standard technologies.
• Metabolic chemical tagging can reveal the full scope of protein lipidation *in vivo*.
• Chemical proteomics will have a deep impact on understanding of protein lipidation.

**Current Opinion in Chemical Biology** 2015, **24**:48–57

This review comes from a themed issue on **Omics**

Edited by **Benjamin F Cravatt** and **Thomas Kodadek**

For a complete overview see the Issue and the Editorial

Available online 15th November 2014

**http://dx.doi.org/10.1016/j.cbpa.2014.10.016**

1367-5931/© 2014 The Authors. Published by Elsevier Ltd. This is an open access article under the CC BY license (http://creativecommons.org/licenses/by/3.0/).

## Introduction

Amongst the hundreds of classes of known protein post-translational modification (PTM) protein lipidation is unique in enabling direct interaction with cell membranes, ranging from constitutive, stable anchors that can withstand multiple rounds of endosomal recycling, to transient membrane binders that permit rapid switching of subcellular localization. Protein lipidation is found in every form of life, and has evolved to its most sophisticated forms in eukaryotes, in which vesicular trafficking pathways and membrane-bound signaling platforms are strongly regulated by lipidated protein families. These PTMs are also important in disease; many of the enzymes involved in installing and processing protein lipidation have been targeted for drug discovery, resulting in a number of clinical trials. However, the complex and incompletely understood substrate specificity of these enzymes, and its intricate interplay with lipid metabolism and disease context, have contributed to a challenging and thus far inconclusive development process. Numerous protein lipidation substrates have been discovered to date, generally through metabolic radiolabeling with lipid precursors, but the full substrate scope has yet to be determined for any of the known types of lipidation. In particular, very few substrates have been validated at endogenous levels in cells, that is, without resorting to substrate overexpression which may in itself influence lipidation levels, and very little is currently known about how changes induced by genetic mutation, disease or drug treatment quantitatively affect protein lipidation across the proteome.

**Figure 1:**
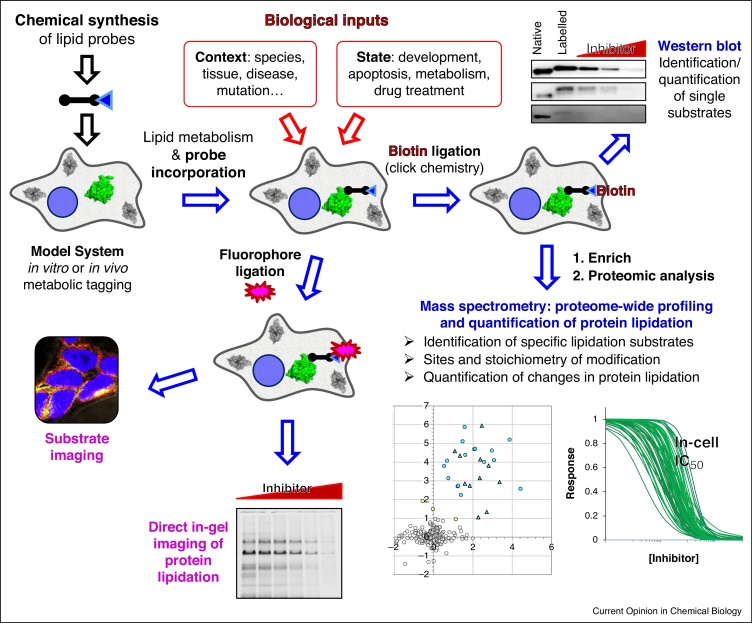
A common workflow can be used to address each of the common classes of lipidation, enabling a wide range of readouts including imaging, pull-downs and mass spectrometry. Rapid profiling and comparisons across experimental conditions or imaging of specific substrates through adaption of the DuoLink proximity rolling-circle amplification technology. The use of selective tool inhibitors and genetic manipulation (e.g. enzyme knockdown or knockout) is used to validate substrates and to quantify the degree of cross-talk between pathways. In combination with stable isotope labeling (SILAC) this approach can be used to quantify changes in lipidation state for proteins across the proteome in response to disease state or drug action, and is applicable to any metabolically active system, including cell lines and *in vivo* models.

Global profiling of protein lipidation lies beyond the range of most standard bioanalytical methods because these relatively large and very hydrophobic PTMs present challenges in protein isolation and separation, restrict ionization of peptides and proteins during mass spectrometric analysis, and are insensitively labeled by radioactive isotopes. Fortunately, protein lipidation is particularly well-suited to analysis through metabolic chemical tagging, since the large size and hydrophobicity of these PTMs facilitates modification with small ‘clickable’ tags whilst avoiding disruption to metabolism and function (Figure 1) [1–3]. These tags can then be addressed either *in situ* or following protein isolation through one of a set of extremely chemoselective reactions that add multifunctional labels exclusively to the modified proteins. Introduction of fluorescent dyes and/or epitope tags allows extremely rapid and sensitive multi-label detection, whereas addition of affinity handles (e.g. biotin) uniquely enables global quantitative analysis of protein lipidation by enrichment coupled to standard liquid chromatography-mass spectrometry. In this review we discuss the development of chemical proteomics technologies that have resulted in the first quantitative whole-proteome studies of the known major classes of protein lipidation, and the first insights into their full scope *in vivo*.

## Protein *N*-acylation at the N-terminus and at lysine

The most-well characterized form of protein *N*-acylation is N-terminal *N*-myristoylation, the irreversible attachment of a C14-fatty acid (myristate) to the N-terminal glycine of substrate proteins, which is catalyzed by *N*-myristoyltransferase (NMT) [4]. NMT is encoded by a single copy gene in lower eukaryotes, whereas in humans and most other higher organisms two NMT genes (*nmt1* and *nmt2*) have been identified. Protein *N*-myristoylation increases affinity for membranes and is required for viability and survival in every organism in which its essentiality has been studied. Dysregulation of myristoylated proteins has been linked to several diseases and NMT has been proposed as a potential drug target in viral, fungal, bacterial or parasitic infections, as well as in cancer [4]. Chemical tools have been developed to study *N*-myristoylation, including alkyne and azido-tagged analogues of the natural lipid substrate (YnMyr and AzMyr, respectively) [5], as well as competitive inhibitors of the protein binding site of NMT. YnMyr is used in most recent studies as it is known to give minimal background labeling [6], and alkyne-tagged lipids appear to recapitulate endogenous lipid metabolism [7]. Potent NMT inhibitors have also been reported, for example against NMT from yeast [8] and from *Trypanosoma brucei* (the causative agent of human sleeping sickness) [9,10], although these had variable selectivity against NMT from various species. More selective inhibitors have been reported recently [11], and can be used as selective chemical tools to pharmacologically knockdown *N*-myristoylation in different organisms. However, metabolism (e.g. chain elongation) of *N*-myristoylation probes can result in trafficking into unrelated lipidation pathways including GPI anchors and *S*-palmitoylation (see following sections). In this context, the combination of NMT inhibitors with YnMyr and quantitative proteomic analysis has proven particularly powerful in establishing the *N*-myristoylated proteome *in vivo*, without interference by off-target protein labeling. In this approach, the response of YnMyr-tagged proteins to selective NMT inhibitors is quantified, and correlated with the identification of each protein as a substrate or non-substrate of NMT.

In the first study of this type, this technology was applied to live malaria parasites and identified about 30 NMT substrates across the proteome, the large majority for the first time, highlighting the potential for *de novo* substrate identification [12^••^]. YnMyr labeling was also used to demonstrate that NMT inhibitors acted on-target in live parasites, and to validate NMT as an antimalarial drug target. A further refinement used chemical proteomic tools that enabled direct identification of the site of *N*-myristoylation, resulting in direct identification of the co-translationally and post-translationally *N*-myristoylated proteomes of human cells using a NMT inhibitor combined with quantitative chemical proteomics [13^••^]. More than 100 NMT substrates were directly identified in this study, >90% for the first time at endogenous protein levels, along with quantitative in-cell IC_50_ inhibition profiles for most of these proteins. Notably, monitoring myristoylation during induction of apoptosis identified 40 substrates that are *N*-myristoylated post-translationally at an internal site, mainly following caspase cleavage, and these proteins may have a specific role in mediating this important cellular process. In the future, a similar approach could be applied to establish the substrate specificity of the NMT1 and NMT2 isozymes in human cells.

**Figure 2:**
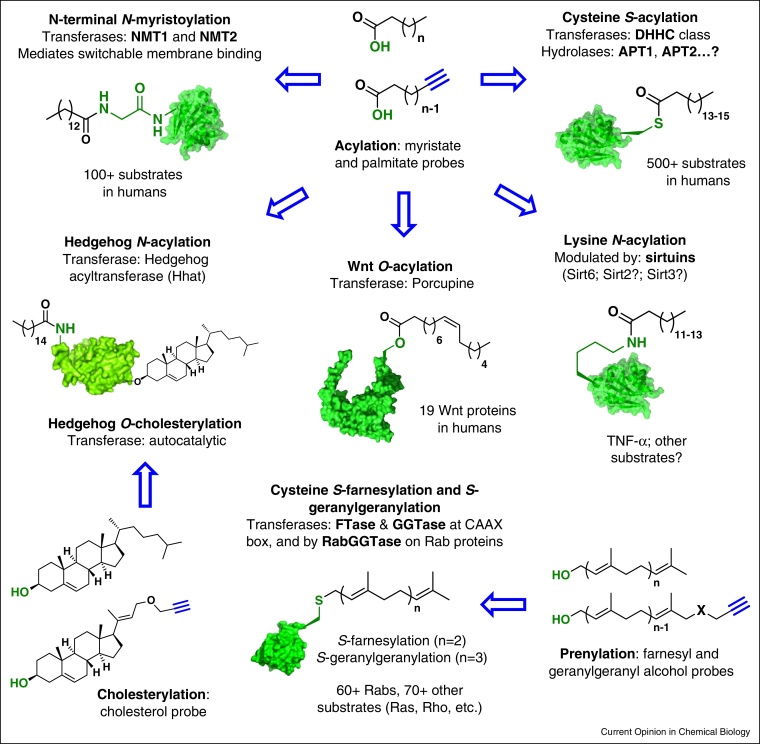
Structures and known scope of the major forms of protein lipidation. Each type of lipidation is involved in important disease pathways in addition to basal cellular function and development, and all types can be profiled in cells using the common technology platform outlined in Figure 1, for the discovery and elucidation of substrate scope and function.

The context of human infection recently provided the first example of reversal of N-terminal *N*-myristoylation; in this study, enzymatic treatment of YnMyr-tagged cell lysates revealed that the *N*-myristoylglycine moiety can be hydrolyzed by a secreted bacterial effector protein with cysteine protease activity, the *Shigella* virulence factor IpaJ [14^•^]. This process is itself irreversible since the N-terminal glycine is also cleaved from the protein, and allows *Shigella* to exploit host trafficking pathways during bacterial infection. In the future, IpaJ may also prove a useful and complementary tool for analysis of *N*-acylation, although its substrate scope has yet to be determined in cells (Figure 2).

*N*-Acylation is also known to occur at the N-terminal cysteine of the hedgehog (Hh) protein family; Hh signaling is mostly inactive in healthy adults but is reactivated in various cancers, and the Hh pathway is a widely studied anticancer drug target with many inhibitors in clinical trials (see also protein cholesterylation, below) [15]. Acylation is catalyzed by a Hh-specific enzyme, hedgehog-acyltransferase (HHAT), a multi-pass transmembrane protein in the membrane bound O-acyltransferase (MBOAT) family. Whilst the large majority of MBOATs transfer lipids to hydroxyls during lipid processing (and in a few cases to proteins, see *O*-acylation), HHAT *S*-palmitoylates Hh proteins at an N-terminal cysteine; this initial thioester rapidly rearranges through *S*-to-*N* acyl shift to produce the mature N-terminal *N*-palmitoyl Hh [16]. Hh *N*-palmitoylation is an excellent target for chemical tagging with azide or alkyne-tagged analogues, and several studies have used this approach to date to demonstrate the essentiality of HHAT and its role in Hh signaling [16,17^••^]. A putative cell-active tool inhibitor for HHAT was also recently reported [18], although its selectivity and stability remain to be proven *in vivo*.

Long-chain fatty acids including myristic acid have also been reported at lysine side-chains in a process that is thought to be independent of NMT, for example on interleukin 1 alpha and tumor necrosis factor (TNF) alpha [19,20]. A combination of biochemical experiments and alkyne-tagged fatty acid labeling experiments was used to explore the function of this post-translational modification, and NAD-dependent protein deacetylase sirtuin-6 (SIRT6) was shown to hydrolyze myristoyl and possibly other long chain acyl moieties on specific residues of TNF-alpha, which regulates the secretion of TNF-alpha [21^••^]. Subsequently, a wide range of sirtuins was shown to have long chain *N*-acyllysine deacylating activity in an isolated enzyme system [22,23]. The enzyme(s) that may act as transferases in this process have yet to be identified, and there remains the possibility that the phenomenon is the result of non-specific attack by reactive acyl-CoA precursors [24]; in this view, the sirtuins may mediate a damage limitation mechanism, with a co-evolved regulatory effect on protein function for certain substrates. Furthermore, given the very broad substrate range of the sirtuins *in vitro*, there is an emerging consensus that their roles can only be determined *in vivo*, which will require more selective Sirt inhibitors and advances in chemical proteomic technology to identify sites of *N*-acylation. Further studies are also needed to identify any enzymes that may be involved in incorporation of long-chain fatty acids on lysine side-chains.

## Protein *S*-acylation at cysteine

*S*-Acylation occurs through a thioester linkage at cysteines, and is regulated through acylation by protein acyltransferases (PATs) and removal by a small number of broad-spectrum acyl-protein thioesterases (APTs) [25,26^••^,27]. The major chain is thought to be C16:0 and thus this modification is often termed *S*-palmitoylation, but other chain types are also known and specific determination of chain length or saturation state is very rarely performed due to challenges of analysis. In addition, non-enzymatic chemical *S*-acylation is very likely to occur to a significant extent based on the availability of acyl-CoA in the cell, although this route remains poorly characterized, and by analogy to the sirtuins (see *N*-acylation) it is plausible that a major role for the APTs is the constitutive repair of this metabolic damage [24]. Long-chain *S*-acylation is widespread in eukaryotes, and there are upwards of 500 *S*-acylated proteins known in humans; furthermore, the modification state of a given protein is typically not uniform, allowing regulation of localization and activity. Enzymatic *S*-palmitoylation is predominantly performed by DHHC-motif containing PATs (DHHCs), which are implicated in disease states including Alzheimer's disease and cancer [28]. To date, there is no potent or selective inhibitor available for the DHHC class. 2-bromopalmitate (2BP) is often erroneously deployed as a pan-DHHC tool inhibitor, despite longstanding evidence that its effects are mediated largely through disruption of lipid metabolism [29]. Recent chemical probe studies have demonstrated that 2BP covalently modifies *upwards of 450 proteins* only a few of which are DHHCs [30^•^,31], strongly implying that 2BP should *not* be employed in the study of *S*-palmitoylation. In contrast, a series of recently described selective APT inhibitors [32,33] serve as very useful tools for *S*-palmitoylation studies, extending to applications *in vivo* [34^•^].

*S*-Acylation is most often studied through ‘cysteine-centric’ approaches, where acyl groups are exchanged for reporters, or ‘acyl-centric’ approaches, using metabolic incorporation of chemically tagged acyl chains [26^••^]. ‘Cysteine-centric’ approaches, including acyl-biotin exchange (ABE [35]) and acyl-resin assisted capture (acyl-RAC [36]), will detect any base-labile thiol modification (including *S*-acylation) in cell lysates, and cannot distinguish between these modifications. Here, free cysteines are capped with thiol reactive reagents and modified cysteines revealed though hydroxylamine hydrolysis, for reaction with thiol-reactive biotin analogues or resins. Recent reports in the application of cysteine-centric approaches include identification of palmitoylated superoxide dismutase (SOD1, important in protecting cells from oxidative damage) in endothelial cells [37], and profiling of potentially palmitoylated proteins in adipocytes and adipose tissue [38]. Since this methodology improves detection by liquid chromatography–coupled mass spectrometry by removing the lipid from specifically modified peptide, the site of palmitoylation can sometimes be determined. Although initial efforts in this direction have resulted in modest coverage of up to 170 sites among 400 proteins [36,39], it should be expected that further optimization of proteomic workflows will soon enable whole-proteome analysis of site occupancy by *S*-acylation.

Weaknesses of the cysteine-centric approach include inability to positively identify the modification (since it is lost during analysis), a high false positive rate from background cysteine reactivity, and limited time resolution for dynamic palmitoylation. Direct metabolic incorporation of chemically tagged palmitate is an alternative acyl-centric approach that enables facile pulse-chase quantification of dynamic and static *S*-acylation [26^••^], but is subject to fluctuations in lipid processing, and incubation with a relatively high concentration of tagged lipid may influence metabolic state. However, a recent report demonstrated that a combination of acyl-centric and cysteine-centric approaches can provide enhanced confidence in assigning targets of *S*-acylation [40^••^]. In the major human malaria parasite, *Plasmodium falciparum*, the authors revealed both dynamic and stable *S*-acylation across more than 400 proteins, including key factors in disease. This combined approach is well-suited to any system amenable to culture *in vitro*, and should be adopted by default if maximum confidence is a primary concern.

## Protein *S*-Prenylation at cysteine

Protein *S*-prenylation, the attachment of a farnesyl (C15) or geranylgeranyl (C20) isoprenoid, occurs *via* a thioether bond on cysteine residues, typically near the C-terminus of target proteins. Farnesyl transferase (FTase) and geranylgeranyl transferase type 1 (GGTase-1) prenylate C-terminal CAAX motifs, whereas Rab geranylgeranyl transferase (RabGGTase/GGTase-2) attaches one or two geranylgeranyl groups to a variety of cysteine-containing sequences specifically in Rab proteins, and requires the accessory proteins Rab Escort Protein 1 or 2 (Rep1/2). Protein prenylation is widely conserved in eukaryotes, and substrates include the large Ras, Rho and Rab families of GTPases, nuclear lamins as well as a number of kinases and phosphatases. In addition, certain viral [41] and bacterial effector [42] proteins are known to be prenylated by the host cell upon infection. Prenylation has been widely studied as a drug target in cancer [43] and progeria [44], with prenyl transferase inhibitors (PTIs) entering more than 70 clinical trials [45]; as a result, a plethora of inhibitor classes is available for these enzymes, with the notable exception of RabGGTase for which a highly selective and potent inhibitor has yet to be fully validated in cells [46]. To date the performance of PTIs in the clinic has been limited at least in part due to specific inhibition driving abnormal and compensatory prenylation by the other prenyltransferases. The wide range of PTIs used as tools in cell biology studies raises a challenge in interpretation and reproducibility, since the potency and selectivity of most of these inhibitors has not been established in a relevant cellular context. As isoprenoids are intermediates of the mevalonate pathway, prenylation is also inhibited by statins (HMG-CoA reductase inhibitors) and this is thought to contribute to the therapeutic effects of this class of drugs [47].

Over the years a large number of chemical reporters to study prenylation have been reported, with recent examples incorporating fluorophores [48], affinity handles [49] or chemical tags for bioorthogonal ligation [50,51]. Such analogues lend themselves to two distinct applications: *in vitro* prenylation of purified proteins or in cell lysates, typically using exogenous recombinant prenyltransferase, or in-cell experiments through metabolic labeling.

*In vitro* prenylation has been used by our lab and others to study the misprenylation of Rabs in models of Choroideremia, a disease resulting from the genetic deletion of Rep1 [51,52] in which unprenylated Rabs accumulate in the eye, leading to retinal degeneration and ultimately blindness. The rate of prenylation of various Rab proteins in lysates was also used to establish cell-free prenylation efficiency for different members of the Rab family [52]. Although in-lysate prenylation is suited to systems currently inaccessible to metabolic labeling (e.g. patient samples and animal models) it is technically complex to implement and relies on identifying proteins that were *not* prenylated. As such any information on what substrates are prenylated *in vivo* can only be inferred; in particular, cross-talk between different types of prenylation during PTI treatment cannot be recapitulated. Secondly, the method is restricted to studying non-equilibrium systems, since it requires an abundance of non-prenylated proteins; in practice, this is often achieved using disruptive inhibition of the mevalonate pathway by statins. Finally, recombinant rat RabGGTase is most commonly used, and may confer subtle differences over the human enzyme; rat Rep2 is also not well-characterized, which throws some doubt on using rat RabGGTase to study Choroideremia.

As noted above (*N*-acylation), live-cell metabolic labeling is particularly powerful for assessment of in-cell potency and target specificity of transferases, and *de novo* discovery of lipidated proteins. Here, the isoprenol analogue is used since the pyrophosphate has limited cell permeability. Conversion to the pyrophosphate *in situ* renders labeling efficiency dependent on a rescue pathway separate from the standard isoprenoid biosynthetic pathway, the activity of which is poorly characterized and varies between cell types. Statin treatment can be used to deplete the endogenous pool of isoprenoids and thus upregulate probe incorporation, but can be strongly disruptive due to concurrent inhibition of cholesterol biosynthesis. A recent study elegantly addressed regulation of isoprenoid uptake through the rescue pathway by means of quantitative mass spectrometry and farnesyl analogues, highlighting the importance of considering metabolism when designing probes and interpreting the data obtained from studies with chemical reporters [53^•^]. An alkyne-tagged isoprenoid analogue has been used to study prenylation in bacterial and viral infection, applying a metabolic labeling strategy to identify prenylation of *Legionella pneumophila* effector proteins by the host prenylation machinery during intracellular infection [42], and revealing the role of prenylation of the long isoform of Zinc finger antiviral protein (ZAP) in the antiviral activity of this protein [54^••^]. Given the broadening range of reported substrates, careful characterization of the scope of prenylation in relevant disease models will be required to realize the genuine therapeutic potential of PTIs in the clinic. Metabolic labeling with a 2D gel imaging strategy was employed to identify targets of a farnesyltransferase inhibitor (FTI) [55]; whilst a small set of differentially prenylated proteins were identified at a single FTI concentration, the use of 2D gels introduces technical limitations in reproducibility, sensitivity, target identification and robust quantification. Unfortunately, the analogue used in most studies to date bears an artificial ether linkage, and studies to date suggest that it fails to differentiate between prenyl chain lengths and may result in non-native membrane distribution of substrates, suggesting it is insufficiently biomimetic to probe the consequences of PTI. However, in future the full vista of *S*-prenylation could be opened up through a combination of improved prenyl analogues and quantitative gel-free metabolic labeling technologies previously successfully applied to *N*-myristoylation and *S*-acylation [12^••^,13^••^,25,26^••^].

## Glycosylphosphatidylinositol (GPI) anchored proteins

Glycosylphosphatidylinositol (GPI)-anchored proteins are an abundant class of glycolipid-bearing cell surface proteins that provide one of the most important cellular machineries for extracellular communication in higher eukaryotes. GPI-anchored proteins are also implicated in many diseases including cancers, prion diseases and several parasitic infections [56–58]. Although bioinformatics methods (e.g. PredGPI) can suggest potential GPI targets [59], experimental approaches for selective and quantitative profiling of modified proteins at a proteome-wide scale are limited. A recent study provides the first reported example of PTM-directed enrichment of GPI-anchored proteins through metabolic chemical tagging of the GPI lipid anchor [12^••^]. Exploiting the promiscuity of cellular fatty acid processing machineries, incubation of YnMyr with the malaria parasite *P. falciparum* led to metabolic labeling of NMT substrates (see above) and also GPI-anchored proteins, the latter including key mediators of immunogenicity and potential vaccine targets. A simple base-treatment prior to affinity enrichment was sufficient to distinguish amide-linked *N*-myristoylation from ester-linked GPI *O*-myristoylation, and led to the identification of all known and several novel GPI-anchored proteins. This approach should prove applicable to global GPI protein profiling in other (e.g. human) systems.

## Protein *O*-cholesterylation

Protein cholesterylation has so far been observed only in the hedgehog (Hh) family of secreted proteins, which undergo posttranslational autocleavage of their C-terminal domain with concomitant *O*-cholesterylation at the C-terminal acid. Hh proteins are key players in embryonic development, stem cell maintenance and tissue repair, and as noted above are aberrantly overexpressed in several cancers [15]. Although the effects of loss of cholesterylation are readily modeled by deletion mutants, many questions concerning the role of intact wild-type cholesterylation remain unanswered due to the lack of robust tools to study the modification in living cells and in live organisms. The first report of chemical tagging of cholesterylation focused on the most studied member of the human Hh family, sonic hedgehog (Shh), and used an azide-tagged cholesterol analogue in a cell line [60]; whilst labeling was demonstrated, low efficiency and toxicity limited the scope of questions that could be addressed. The most recent addition to the protein cholesterylation toolset is a panel of alkyne-cholesterol analogues displaying far superior performance to previous probes [17^••^], achieving efficient labeling at low probe concentration and no cellular toxicity. An optimal probe provided quantitative profiling of cholesterylation in multiple pancreatic cancer cell lines with elevated Shh expression, the first direct evidence for extensive Shh cholesterylation in secreted multimeric signaling complexes, confocal fluorescent imaging of labeled Shh in human cells, and visualization of cholesterylated Hh proteins in zebrafish embryos. It is anticipated that in future these chemical tools will shed more light on the roles of cholesterylation in secretion and in the context of developing organisms.

## Perspectives

Rapid progress has been made over the past few years in our understanding of the global scope and potential druggability of protein lipidation, due in large part to the development of quantitative chemical proteomic technologies that can meet the challenge of analyzing these large and hydrophobic PTMs. The combination of tagging with selective inhibitors or other complementary approaches has proven particularly powerful, and can further provide unique insights into in-cell inhibitor target engagement. In the near future, several important aspects of protein lipidation biology are ripe for further development.

*Enhancing bioinformatic predictions*: new chemical proteomics tools for the direct analysis of the sites of protein lipidation *in vivo* offer the opportunity to improve bioinformatic prediction algorithms, which currently rely on very limited learning sets [12^••^,13^••^].

*Broadening scope*: tagging methodologies offer a unique approach to identifying lipidation at amino acid side chains beyond *N*-linkage and *S*-linkage, and further integration with advanced mass spectrometry analysis should enable routine profiling of O-acyl and alkyl side chains. For example, *O*-palmitoleoylation (16:1) of Wnt proteins by the MBOAT family protein Porcupine (Porc) is known to be critical for Wnt signaling, and has been recognized as a druggable node in the context of cancer [61].

*Prospective PTM discovery*: the discovery of the first substrates of myristoylation, palmitoylation, farnesylation and geranylgeranylation was achieved through radiolabeling; given the notoriously poor sensitivity of this approach and historic limitations of proteomics, it is perhaps unsurprising that these are among the most abundant classes of protein lipidation in the cell. Robust tag-enrichment technologies now present the opportunity to systematically profile metabolic incorporation of novel lipids across the proteome, for *de novo* discovery of PTMs previously overlooked due to their rarity or mass spectrometric intractability.

*Protein lipidation in disease models*: as noted above, the first proteome-wide studies of lipidation in pathogenic bacteria and parasites in a relevant host context have recently been reported [40^••^,42], and there is much scope for the application of these tools to study host/pathogen interactions in relevant models of viral infection. Bacterial lipoproteins, which carry an *S*-linked diacylglycerol motif, have previously been probed using tagging approaches in *Escherichia coli* [62]; optimized quantitative methods should be widely applicable in a variety of pathogenic bacteria, to further illuminate the functional roles of this key bacterial machinery in virulence.

*Interplay with lipid metabolism*: lipid metabolism is known to be dysregulated in many cancers and as a consequence of therapy (e.g. statin or fatty acid synthase inhibitor treatment), and there is recent evidence that tissue-specific lipid metabolism directly impacts the profile of protein lipidation [63]. The potential for incorporation of branched lipids, unsaturated fatty acids and cholesterol-related hormones remains almost unexplored at present, and the combination of lipidomics with tagging approaches, as recently explored for prenylation [53^•^], is likely to reveal a complex interplay between these systems.

*Imaging specific protein lipidation*: the widespread nature of lipids in the cell, both in membranes and on proteins, renders global analysis by cellular imaging of limited utility; even in an ideal case, only the overall distribution of lipids and/or lipidated proteins is revealed [64]. An exception is cholesterylation which appears to be uniquely attached to Hh proteins; following clearance of membrane lipids, this modification can be imaged with good fidelity [17^••^]. In the first step towards a more general methodology, Gao and Hannoush, recognizing that substrate-specific imaging requires protein identity coupled to covalent modification by a lipid, employed a combination of palmitoyl tagging and specific antibodies coupled to oligonucleotides, enabling proximity-directed detection by rolling-circle amplification [65]. Preliminary studies suggest that with optimization this rather complex approach is capable of direct detection of palmitoylation of Wnt, Hh and Ras proteins [66^••^], but significant technological hurdles remain if this approach is to be rendered generally applicable, or of use in live cell imaging.

The unique PTM-driven, system-wide profiling capabilities of chemical tagging can now be applied in a great variety of contexts in which the role of protein lipidation is currently unknown due to the historical lack of tools with which to ask key questions: Which substrates are modified, and where and when does this occur? Which enzymes and proteins write, erase and read the modification, and how are they regulated? To what extent is lipidation dysregulated in a pathological context, and where do the therapeutic opportunities lie? It is remarkable that most of these questions have not yet been systematically addressed in relevant systems, despite enormous investment in development of potent and selective inhibitors for several of these pathways, above all against protein prenylation. It is essential that we understand the global scope and dynamic range of this complex and widespread class of PTMs before we can unlock the full therapeutic potential of protein lipidation.

## References and recommended reading

Papers of particular interest, published within the period of review, have been highlighted as:

• of special interest
•• of outstanding interest
